# Investigation on the Electrochemical Micromachining of Micro Through-Hole by Using Micro Helical Electrode

**DOI:** 10.3390/mi11020118

**Published:** 2020-01-21

**Authors:** Baohui Liu, Hang Zou, Haixuan Luo, Xiaoming Yue

**Affiliations:** 1 School of Mechanical Engineering, Shandong University, Jinan 250061, China; LBHui@mail.sdu.edu.cn (B.L.); 201700162058@mail.sdu.edu.cn (H.Z.); 201700162073@mail.sdu.edu.cn (H.L.); 2 Key Laboratory of High efficiency and Clean Mechanical Manufacture, Ministry of Education, Shandong University, Jinan 250061, China; 3 Suzhou Research Institute, Shandong University, Suzhou 215123, China

**Keywords:** electrochemical micromachining (EMM), micro through-hole, micro helical electrode, stray corrosion, non-conductive mask

## Abstract

The instability of machining process caused by the difficulty of the electrolyte refresh in electrochemical micromachining (EMM) of micro through-hole has been an unsolved problem. Thus, this paper investigates the electrochemical micromachining of micro through-hole by using a micro helical electrode combining with the jetting electrolyte. With the help of high-speed rotation of micro helical electrode and its spiral shape, the internal electrolyte can be stirred while the external jetting electrolyte can flow into the hole along the spiral groove to refresh the electrolyte effectively, thereby, improving the machining stability of EMM. Firstly, the influence of the process parameters on the fabrication of micro through-hole in the EMM by using micro helical electrode without non-conductive mask is investigated. Based on the optimization of the process parameters, a micro through-hole with an inlet dimension of 121.6 μm and an outlet dimension of 114.9 μm is obtained successfully. Furthermore, this paper also tries to use the micro helical electrode coated with the non-conductive mask to decrease the bad influence of the stray corrosion attack. It is found that the non-conductive mask coated on the surface of micro helical electrode can improve the machining accuracy significantly under the condition of low pulse frequency (≤1 KHz). However, its good effect on preventing the stray corrosion decreases along with the increase of the pulse frequency.

## 1. Introduction

As one of the widely used non-traditional machining methods, electrochemical machining (ECM) is an anodic dissolution process, in which the anode material can be removed through the ion dissolution based on the Faraday’s law when a sufficient voltage is exerted between the anode electrode and cathode workpiece in which the gap is filled with the electrolyte [1]. Furthermore, ECM technology has many unique advantages such as no electrode wear and machining force, absence of residual stress, good surface quality, and the machining ability of metal materials regardless of their strength and hardness [2,3]. Thereby, ECM has been widely used to fabricate some special difficult-to-machine components, such as the jet engine turbine blades, steam turbine blades, etc. Also, ECM can be used in the mirror-finishing machining [4,5], whose surface quality even can be comparable with the mechanical polishing [6]. Recently, with the development of microelectromechanical systems (MEMS) and other small precise parts, the demand for micro machining is constantly increasing. Because of the above unique advantages, ECM has been attracting more and more researchers’ attention on the machining of micro parts, which is called electrochemical micromachining (EMM) [7], a variant of traditional ECM.

Specifically, among all the micro structures, micro holes are one of the most important and useful micro structures in many kinds of components and parts such as the cooling holes in the jet turbine blades, the microfluidic channel in micro reactors and micro exchangers, and the fuel injection nozzles in the engine, etc. [8,9]. Thus, nowadays, fabrication of micro holes on the parts with extremely hard and tough materials by EMM is attracting more and more researchers’ attention [10,11]. However, EMM also faces many challenges in the machining of micro holes with high quality and precision. The biggest challenge is unstable machining process because the electrolyte refresh in quite narrow space is extremely difficult during the machining of micro hole by EMM, causing the accumulation of electrolysis products, such as the bubble and metal hydroxide. As a result, the short circuit occurs frequently and the machining cannot go on normally. Usually, in the machining of macro hole by traditional ECM, the internal electrolyte in the gap is enforced to refresh to guarantee the process stability, such as the hollow electrode [1]. However, the above method is invalid in EMM because it is difficult to fabricate the hollow structure in a micro electrode with a diameter of less than 100 μm. The forced circulation of the external electrolyte has no obvious effect because it is difficult for external electrolytes to enter via micro holes through a very small sidewall gap between the tool electrode and hole in EMM.

To solve the difficulty of the electrolyte refresh in EMM, some researchers adopt some assisted means such as the ultrasonic assistance [12] or design the cathode electrode with particular shape such as the helical electrode [13]. In the drilling of micro hole with high accuracy by EMM, using the ultrasonic assistance is not realistic because micro electrode must be rotating and fixed on the rotating spindle. However, it is difficult to install a rotating spindle in the ultrasonic generator. Thus, it is thought that using the helical electrode—which is a standard commercial product—to improve the electrolyte refresh is a simple and effective method in the machining of micro hole by EMM. Tsui et al. [13] investigated the electrochemical micro drilling using the helical electrode and they verified that the shape accuracy of micro hole can be significantly improved by using micro helical tool. In their study, the inlet and outlet diameters of micro hole are 335 μm and 299 μm, respectively using the helical electrode with the diameter of 200 μm under the machining conditions shown in their [13].

To further improve the machining accuracy of micro hole in EMM using micro helical electrode in a simple and low-cost way, this paper combines the high-speed rotating helical electrode with forced circulation of external electrolyte by jetting the electrolyte to the entrance of micro hole. On one hand, with the help of high-speed rotation of micro helical electrode, the internal electrolyte can be stirred and renewed. On the other hand, with the help of spiral shape of micro helical electrode, the external jetting electrolyte can flow into the hole along the spiral groove. The above measures can improve the electrolyte renewal effectively to stabilize the machining process in EMM. Furthermore, this paper also tries to use micro helical electrode coated with a non-conductive mask to decrease the bad influence of the stray corrosion attack. The ultimate objective of this paper is to achieve much higher machining accuracy of micro hole in EMM by a much simpler and lower-cost method.

## 2. Experimental Setup and Details

**Figure 1:**
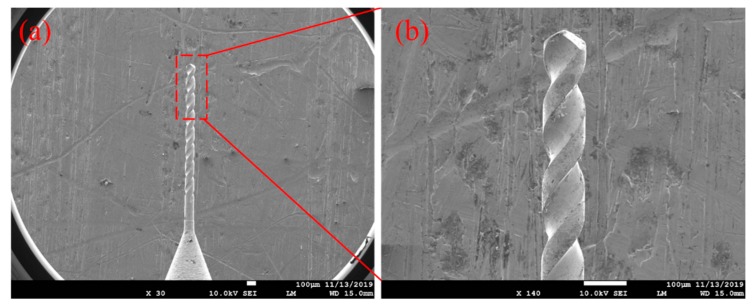
Scanning electron microscope (SEM) image of micro helical electrode (**a**) and enlarged image at the top of micro helical electrode (**b**).

**Figure 2:**
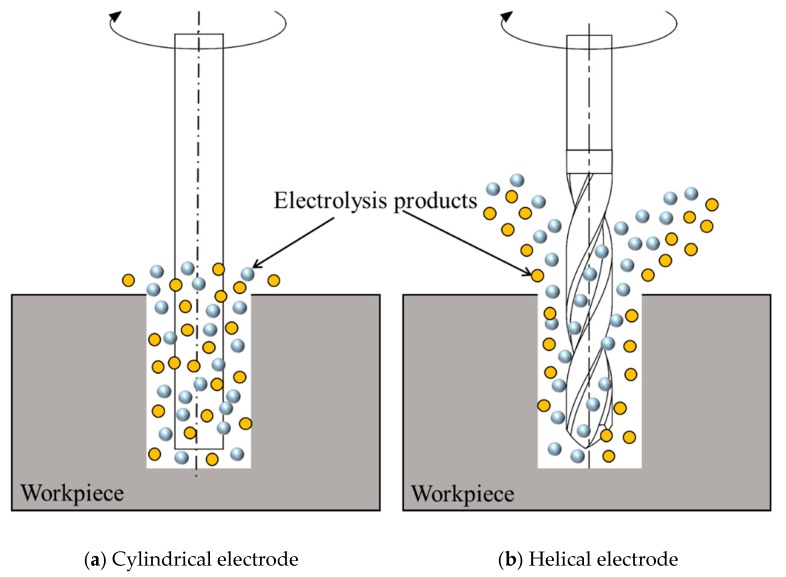
Comparison diagram of cylindrical electrode and helical electrode in transferring the electrolysis products.

In this study, micro helical electrode combined with external jetting electrolyte is used in EMM to enhance the refresh of internal electrolyte inside micro hole. Compared with the traditional cylindrical electrode, micro helical electrode has micro spiral groove structure on its outer surface, as shown in Figure 1. In the EMM of micro hole using micro helical electrode, the shape structure of micro helical electrode can stir the electrolyte and promote the renewal of the electrolyte in the machining zone more strongly than traditional cylindrical electrode, as shown in Figure 2, which has been verified by many researchers [13,14]. Moreover, the flowing electrolyte is jetted to the inlet of micro hole through the needle tube. With the help of micro spiral groove distributed on the outer surface of micro helical electrode, external jetting electrolyte can flow into the machining zone, further improving the renewal of the electrolyte. Micro helical electrode used in this study also has many advantages such as the standardization, seriation, high precision, and low cost. Thus, it is thought that using micro helical electrode combining with the jetting electrolyte is a much simpler and lower-cost way to improve the machining stability and accuracy in the EMM of micro hole.

**Figure 3:**
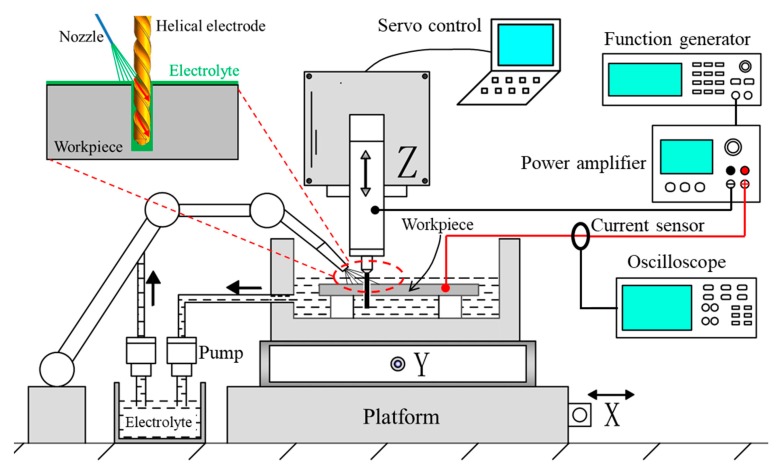
Schematics of experimental setup used to fabricate micro through-hole by electrochemical micromachining (EMM).

Figure 3 shows the schematics of self-developed experimental setup used in EMM. The device consists of a precise XYZ movement stage with the motion accuracy of 0.5 μm, an electrolyte recycling system, a power system, a real-time monitoring system, a servo control system, and other accessories. The high-speed spindle with the radial runout accuracy of 1 μm is installed on the *z*-axis. The micro helical electrode with the diameter of 100 μm is fixed in the collet of the high-speed spindle, which is actually a commercial micro drill. In the power system, the function generator produces the pulse signal and the power amplifier amplifies this signal to required amplitude. The power amplifier is a high-frequency power amplifier ATA-122D whose bandwidth range is DC~24 MHz with the maximum power of 35 W (made by Xi’an Aigtek Electronic Technology Ltd.). The real-time monitoring system is used to measure the machining current and voltage. The electrolyte is jetted to the entrance of micro hole by a needle tube with the flow rate of 30 mL/min whose internal diameter is 410 μm. When using the jetting electrolyte, the workpiece is not immersed in the electrolyte and only the gap between the helical electrode and workpiece is jetted by the electrolyte. The rotating helical electrode is fed along the *z*-axis at a uniform speed in the machining process of EMM. The experimental conditions used in this study are shown in Table 1.

In the experimental setup, a contact perception is developed to determine the relative position between the helical electrode and workpiece, which can be described briefly as follows: a small voltage is applied between the helical electrode and workpiece. Then, every 1 μm the helical electrode moves towards the workpiece surface, the machine tool will detect the voltage between the helical electrode and workpiece. When the helical electrode touches the workpiece surface, the voltage between the helical electrode and workpiece becomes zero and the movement of the helical electrode is stopped by the machine tool immediately. Through the above method, the relative position between the helical electrode and workpiece can be determined preciously.

In this study, two kinds of micro helical electrodes whose material are cemented carbide are used. One is the helical electrode without the non-conductive mask coated on the outer surface and the other is the micro helical electrode with the non-conductive mask coated on the outer surface by the electrophoretic deposition (EPD) technology [15]. The EPD is a process that colloidal particles suspended in a liquid medium migrate under the influence of an electric field (electrophoresis) and are deposited onto an electrode. All colloidal particles that can be used to form stable suspensions and that can carry a charge can be used in electrophoretic deposition. This includes materials such as polymers, pigments, dyes, ceramics, and metals. In this study, the epoxy resin is used as the non-conductive mask.

## 3. Fabrication of Micro Through-Hole Using Micro Helical Electrode without Mask

The machining of micro through-hole in EMM is conducted under the experimental conditions shown in Table 1. The thickness of the workpiece is 100 μm. Firstly, the helical electrode is placed 15 μm above the workpiece surface through the contact perception. Then, the helical electrode feeds down 150 μm to make sure that a through-hole can be obtained in EMM. Every experimental group contains three repeated experiments. After the machining experiment, the diameters of the inlet and outlet of micro through-hole are measured by the optical microscope. The detailed images of micro through-hole are observed by the scanning electron microscope (SEM), respectively. Based on the above measuring results, the process parameters such as the machined voltage, pulse frequency, pulse duty cycle, electrolyte concentration, rotating speed of the helical electrode, and feeding speed on the machining accuracy and quality of micro through-hole in EMM are investigated. Finally, a high-precision micro through-hole is fabricated under the optimized process parameters.

### 3.1. Influence of Machined Voltage on the Machining of Micro Through-Hole

**Figure 4:**
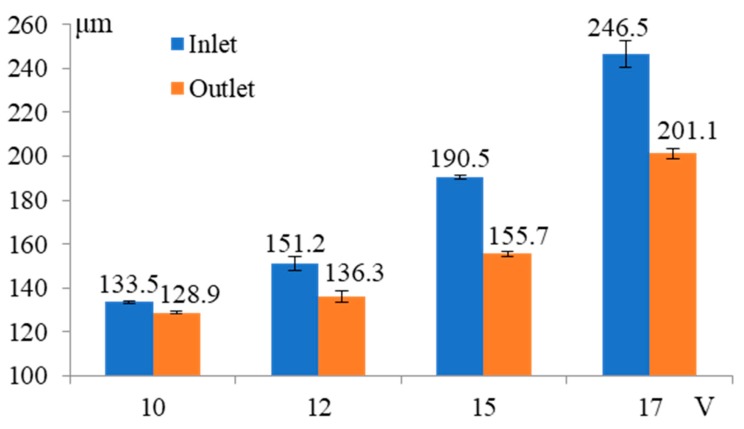
Influence of machined voltage on the inlet and outlet dimension of micro through-hole.

**Figure 5:**
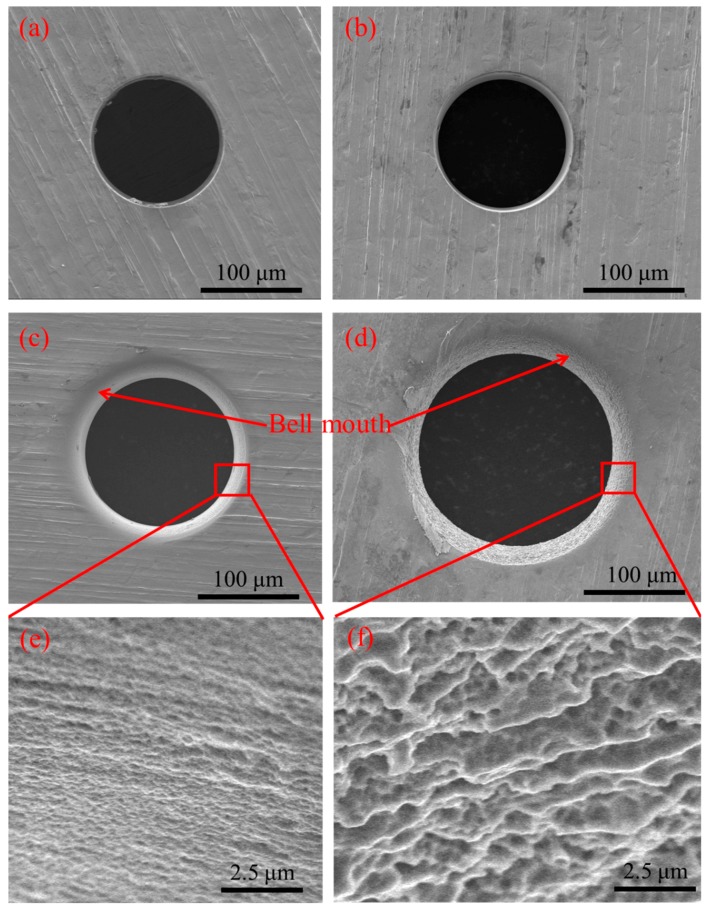
SEM images of inlet profile of micro through-hole fabricated under machined voltages of 10 V (**a**), 12 V (**b**), 15 V (**c**), and 17 V (**d**). (**e**) and (**f**) are partial enlarged images of (**c**) and (**d**).

Figure 4 shows the influence of machined voltage on the inlet and outlet dimension of micro through-hole. Five groups of machining experiments of micro through-holes with the machined voltages of 8, 10, 12, 15, and 17 V are conducted. The other experimental conditions are 2000 rpm rotating speed, 500 kHz pulse frequency, 20% duty cycle, 1 μm/s feeding speed, 2% NaNO_3_ electrolyte, respectively. The machining of micro through-hole with the machined voltage 8 V under the above machining conditions fails due to serious short circuit. The experimental results show that both the inlet and outlet dimension increase rapidly with the increase of machined voltage. Moreover, the difference between the inlet and outlet values, which reflects the taper of micro through-hole, increases significantly with the increase of machined voltage. Figure 5 shows the SEM images of inlet profile of micro through-hole fabricated under different machined voltages. From the figure, it can be found that the stray corrosion attack around the inlet of micro through-hole becomes much more obvious along with the increase of machined voltage, resulting in the bell-mouth profile. Furthermore, the surface quality of hole wall decreases along with the increase of machined voltage, as shown in Figure 5e,f. Thus, it can be concluded that much lower machined voltage should be required for achieving much higher accuracy and quality in the EMM of micro through-hole.

### 3.2. Influence of Pulse Frequency on the Machining of Micro Through-Hole

**Figure 6:**
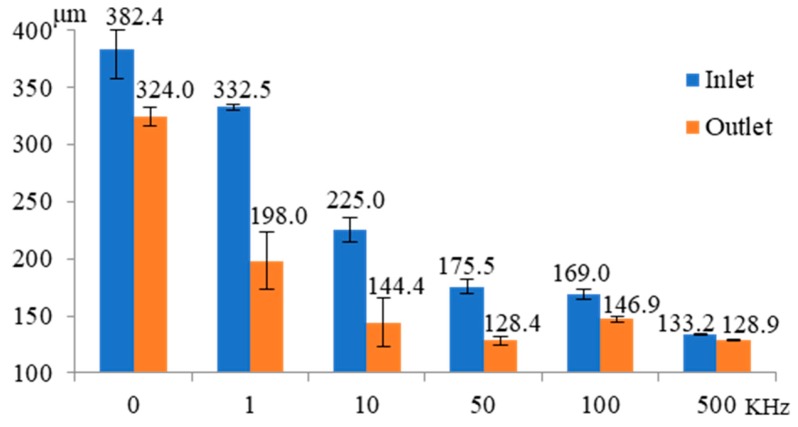
Influence of pulse frequency on the inlet and outlet dimension of micro through-hole.

**Figure 7:**
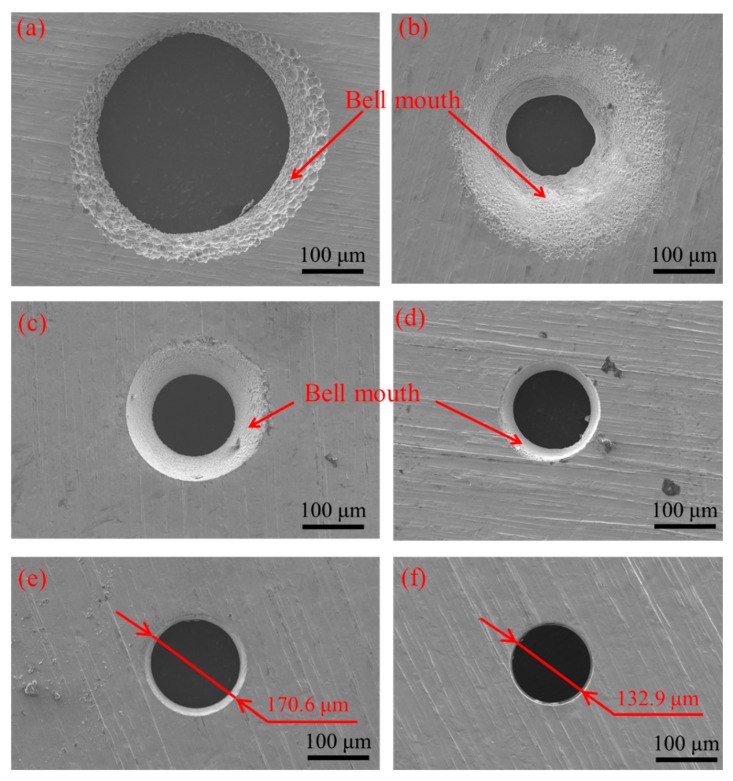
SEM images of inlet profile of micro through-hole fabricated under pulse frequencies of 0 kHz (**a**), 1 kHz (**b**), 10 kHz (**c**), 50 kHz (**d**), 100 kHz (**e**), and 500 kHz (**f**).

**Figure 8:**
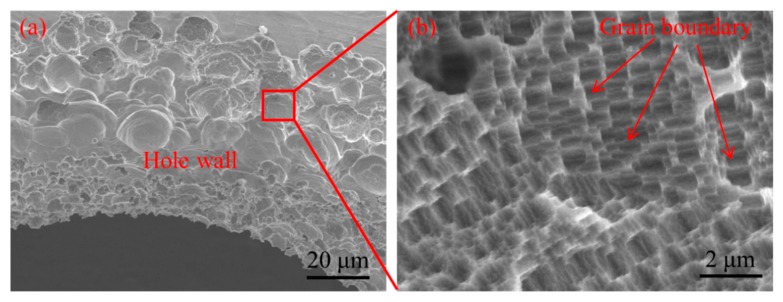
Partial enlarged SEM images around the inlet of micro through-hole fabricated under pulse frequency of 0 kHz.

Figure 6 shows the influence of pulse frequency on the inlet and outlet dimension of micro through-hole. Six groups of machining experiments of micro through-holes with the pulse frequencies of 0, 1, 10, 50, 100, and 500 kHz are conducted. The other experimental conditions are 2000 rpm rotating speed, 10 V machined voltage, 20% duty cycle, 1 μm/s feeding speed, 2% NaNO_3_ electrolyte, respectively. The experimental results indicate that both the inlet and outlet dimension decrease rapidly with the increase of pulse frequency. Moreover, the difference between the inlet and outlet values, which reflects the taper of micro through-hole, also decreases significantly with the increase of pulse frequency. Figure 7 shows the SEM images of the inlet profile of micro through-hole fabricated under different pulse frequencies. From the figure, it can be found that the shape accuracy of micro through-hole increases markedly with the increase of pulse frequency. When the pulse frequency is low, such as 0 KHz and 1 KHz, the shape of micro through-hole is not circular and the bell-mouth profile around the inlet of micro through-hole caused by the stray corrosion attack is quite obvious. Furthermore, the surface quality of the hole wall also increases along with the increase of pulse frequency. Under the machining condition of 0 KHz pulse frequency, the machining surface is seriously bad and the ion dissolution along the grain boundary is clearly visible, as shown in Figure 8. Thus, it can be concluded that a much higher pulse frequency should be used for achieving much better accuracy and quality in the EMM of micro through-hole.

### 3.3. Influence of Duty Cycle on the Machining of Micro Through-Hole

**Figure 9:**
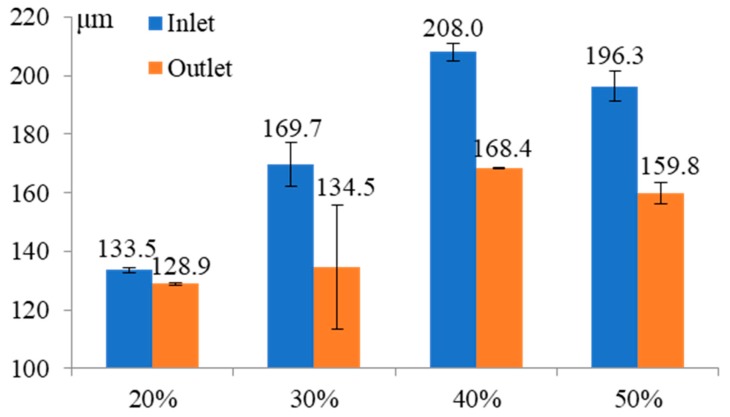
Influence of pulse duty cycle on the inlet and outlet dimension of micro through-hole.

**Figure 10:**
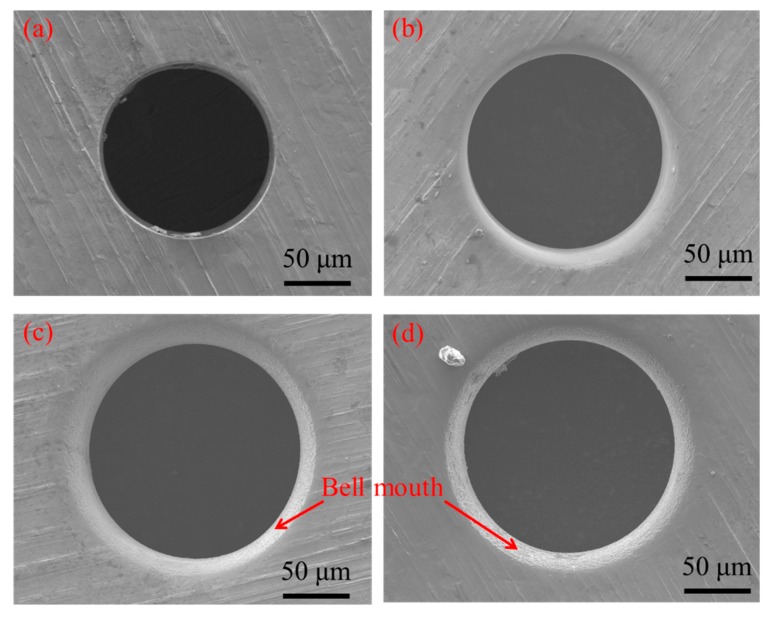
SEM images of inlet profile of micro through-hole fabricated under duty cycles of 20% (**a**), 30% (**b**), 40% (**c**), and 50% (**d**).

Figure 9 shows the influence of pulse duty cycle on the inlet and outlet dimension of micro through-hole. Four groups of machining experiments of micro through-holes with the duty cycles of 20, 30, 40, and 50% are conducted. The other experimental conditions are 2000 rpm rotating speed, 500 kHz pulse frequency, 10 V machined voltage, 1 μm/s feeding speed, 2% NaNO_3_ electrolyte, respectively. The experimental results show that from the overall trend, both the inlet and outlet dimension increase with the increase of pulse duty cycle. Moreover, the difference between the inlet and outlet values, which reflects the taper of micro through-hole, also increases significantly with the increase of pulse duty cycle from the overall trend. Figure 10 shows the SEM images of inlet profile of micro through-hole fabricated under different duty cycles. From the figure, it can be found that the bell-mouth profile around the inlet of micro through-hole caused by the stray corrosion attack is quite obvious with higher duty cycle. Thus, it can be concluded that much lower duty cycle should be used for achieving much better accuracy and quality in the EMM of micro through-hole.

### 3.4. Influence of Electrolyte Concentration on the Machining of Micro Through-Hole

**Figure 11:**
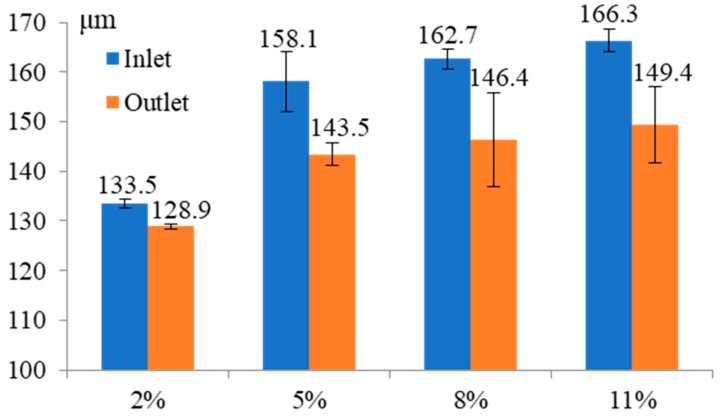
Influence of electrolyte concentration on the inlet and outlet dimension of micro through-hole.

**Figure 12:**
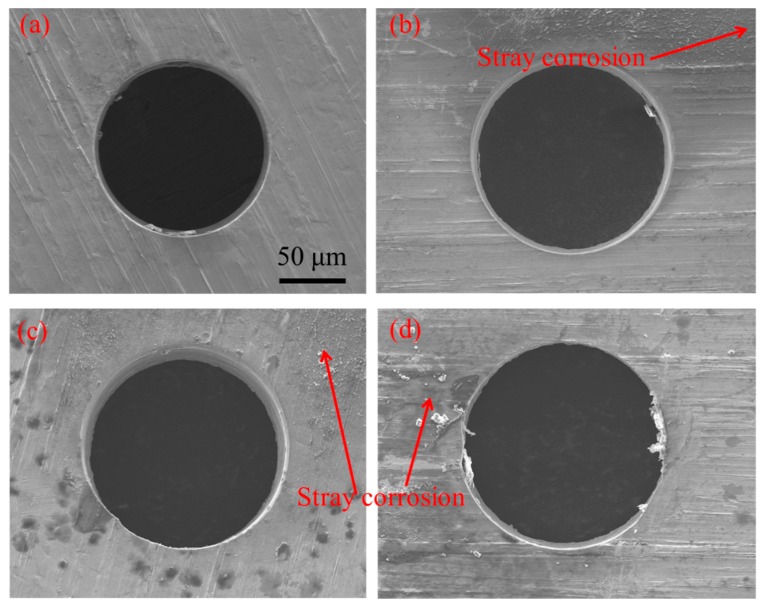
SEM images of inlet profile of micro through-hole fabricated under the electrolyte concentrations of 2% (**a**), 5% (**b**), 8% (**c**), and 11% (**d**).

Figure 11 shows the influence of the electrolyte concentration on the inlet and outlet dimension of micro through-hole. Four groups of machining experiments of micro through-holes with the electrolyte concentrations of 2, 5, 8, and 11% are conducted. The other experimental conditions are 2000 rpm rotating speed, 10 V machined voltage, 500 kHz pulse frequency, 20% duty cycle, 1 μm/s feeding speed, respectively. The experimental results show that both the inlet and outlet dimension increase with the increase of electrolyte concentration. Moreover, the difference between the inlet and outlet values, which reflects the taper of micro through-hole, also increases with the increase of electrolyte concentration. Figure 12 shows the SEM images of inlet profile of micro through-hole fabricated under different electrolyte concentrations. From the figure, it can be found that on the conditions of different electrolyte concentrations, there is no obvious bell-mouth profile generated around the inlet of micro through-hole caused by the stray corrosion attack, which shows that compared with machined voltage, pulse frequency and pulse duty, the electrolyte concentration has less influence on the shape of micro through-hole. However, the stray corrosion attack around the hole inlet becomes much more obvious along with the increase of electrolyte concentration. Thus, much lower electrolyte concentration should be required for achieving micro through-hole with high surface quality.

### 3.5. Influence of Rotating Speed of Helical Electrode and Jetting Electrolyte on the Machining of Micro Through-Hole

In ECM process, metal hydroxide and gas bubbles are generated rapidly in the small gap between the electrodes, which becomes a barrier to the current flow and further machining. Hence, for the continuation of the dissolution process, these reaction products must be removed from the gap quickly and totally. In this study, we try to combine the helical electrode with the jetting electrolyte. To validate the effect of the above method in improving the electrolyte renewal, the following experiments and simulation are conducted.

**Figure 13:**
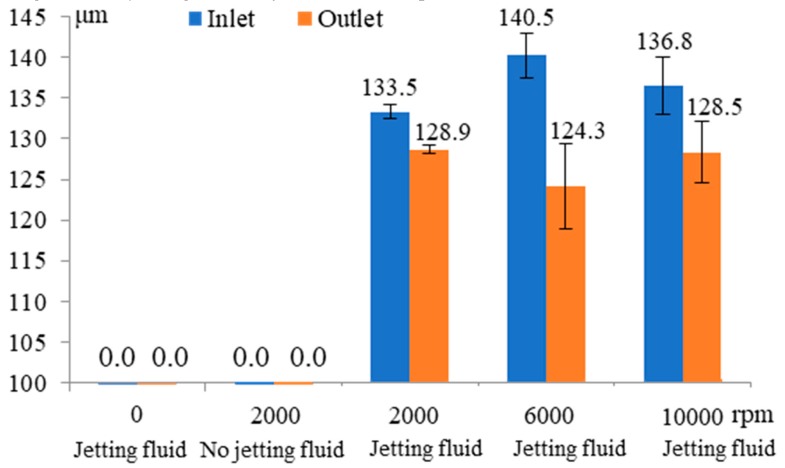
Influence of rotating speed of helical electrode and jetting electrolyte on the inlet and outlet dimension of micro through-hole.

Figure 13 shows the influence of rotating speed of helical electrode and jetting electrolyte on the machining of micro through-hole. Five groups of comparative machining experiments of micro through-holes with the conditions of 0 rpm and jetting fluid, 2000 rpm and no jetting fluid, 2000 rpm and jetting fluid, 6000 rpm and jetting fluid, 10,000 rpm and jetting fluid are conducted. The other experimental conditions are 10 V machined voltage, 500 kHz pulse frequency, 20% duty cycle, 1 μm/s feeding speed, 2% NaNO_3_ electrolyte, respectively. When using the condition of no jetting electrolyte, the workpiece is totally immersed in the electrolyte and the electrolyte is kept stationary. The experimental results show that the machining of micro through-hole with the rotating speed of 0 rpm and jetting fluid under the above machining conditions fails due to the serious short circuit even if the flowing electrolyte is jetted into the entrance of the micro hole. However, when the rotating speed of 2000 rpm and jetting fluid is adopted, the micro through-hole can be fabricated successfully. Thereby, the above experimental results strongly demonstrate the helical electrode with high rotating speed can promote the renewal of the electrolyte and guarantee the stability of the machining process in EMM. However, when the rotating speed of micro helical electrode increases to 6000 rpm and 10,000 rpm, both the inlet and outlet dimensions of micro through-hole does not show obvious changing law, which demonstrates that under the above machining conditions, the rotating speed of 2000 rpm is enough to ensure the renewal of the electrolyte. Moreover, it is also found that the machining of the micro through-hole with the rotating speed of 2000 rpm and no jetting fluid under the above machining conditions fails due to the serious short circuit even if the rotating helical electrode is used. Combined with the above experimental results, it can be concluded that jetting electrolyte to the entrance of hole also plays a significant role in promoting the renewal of the electrolyte. Thus, based on the above experimental results, the rotating speed of 2000 rpm and jetting fluid is adopted for all machining experiments.

**Figure 14:**
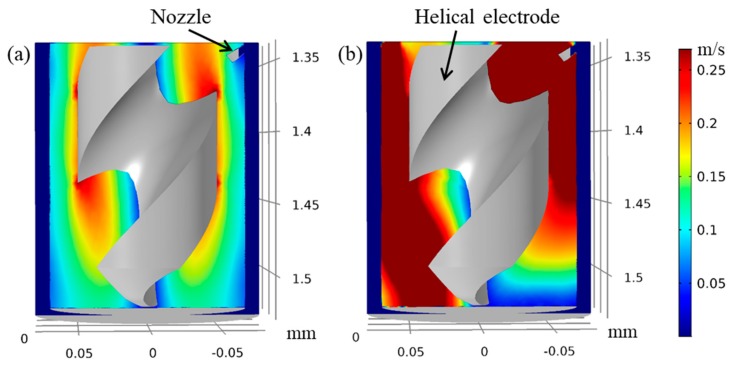
Flow field distribution of electrolyte without jetting electrolyte (**a**) and with jetting electrolyte (**b**).

In order to explain the effect of jetting electrolyte on the refresh of electrolyte inside the hole, a fluid simulation is conducted qualitatively. Figure 14 shows the flow field distribution of electrolyte without jetting electrolyte (0 m/s) and with jetting electrolyte (5 m/s) under 2000 rpm rotating speed of helical electrode. It can be found that when using the jetting electrolyte, the flow field of electrolyte inside the hole is improved significantly compared with the simulation without the jetting electrolyte, demonstrating that the jetting electrolyte also has important effect on the renewal of electrolyte.

### 3.6. Influence of Feeding Speed on the Machining of Micro Through-Hole

**Figure 15:**
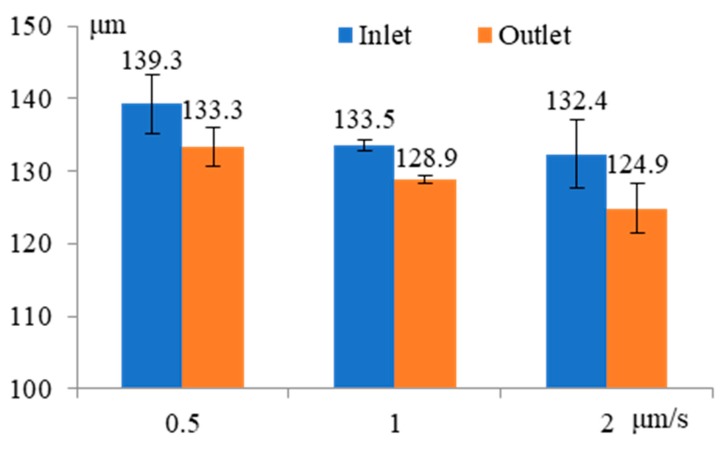
Influence of feeding speed on the inlet and outlet dimension of micro through-hole.

Figure 15 shows the influence of feeding speed on the inlet and outlet dimension of micro through-hole. Three groups of machining experiments of micro through-holes with feeding speeds of 0.5, 1, and 2 μm/s are conducted. The other experimental conditions are 2000 rpm rotating speed, 10 V machined voltage, 500 kHz pulse frequency, 20% duty cycle, 2% NaNO_3_ electrolyte, respectively. The experimental results show that both the inlet and outlet dimension of micro through-hole decrease slightly with the increase of feeding speed. Thus, under the above machining conditions, the feeding speed has quite small influence on the machining accuracy of micro through-hole in EMM.

### 3.7. Fabrication of Micro Through-Hole with High Accuracy by Using Micro Helical Electrode

**Figure 16:**
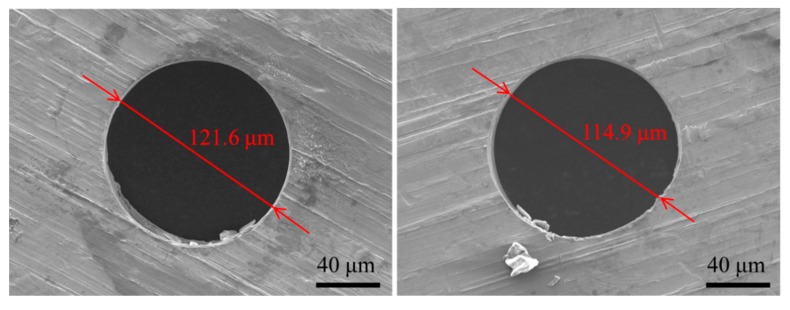
SEM images of inlet and outlet dimension of micro through-hole.

Based on the above experiments of process parameters, the minimum micro through-hole with high accuracy is obtained under the existing machining conditions in our laboratory. The experimental conditions are 2000 rpm rotating speed, 10 V machined voltage, 700 kHz pulse frequency, 20% duty cycle, 1 μm/s feeding speed, 2% NaNO_3_ electrolyte, respectively. Figure 16 shows the SEM image of micro through-hole machined under the above experimental conditions. The inlet and outlet dimensions of micro through-hole are 121.6 μm and 114.9 μm, respectively. The taper of micro through-hole is 1.92°. After the optimization of process parameters, the machining accuracy and quality is significantly improved in the EMM of micro through-hole, demonstrating that using a micro drill combined with the jetting electrolyte is a much simpler and lower-cost way to improve the machining stability and accuracy in the EMM of micro through-hole.

## 4. Fabrication of Micro Through-Hole Using Micro Helical Electrode with Mask

From the above experimental results, it can be seen that although the machining quality and shape of micro through-hole can be easily guaranteed, the dimensional accuracy of the inlet and outlet is still hard to obtain stably due to the stray removal. It is well known that the stray removal or overcut caused by the stray corrosion attack is the biggest problem in the ECM. One solution is to design and optimize the shape and structure of the cathode electrode. The other solution is to coat a non-conductive mask on the surface of the cathode electrode to decrease the bad effect of the stray current. In this section, the outer surface of micro helical electrode is coated with the epoxy resin by the electrophoretic deposition (EPD) technology. The thickness of the non-conductive mask is about 10–20 μm. Thus, in this section, we also try to use the non-conductive mask coated on the surface of micro helical electrode to further improve the machining accuracy of the micro through-hole.

**Figure 17:**
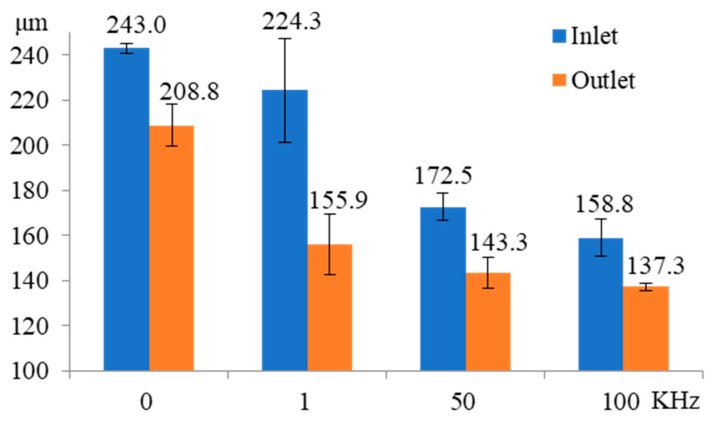
Influence of pulse frequency on the inlet and outlet dimension of micro through-hole fabricated by using micro helical electrode coated with non-conductive mask.

**Figure 18:**
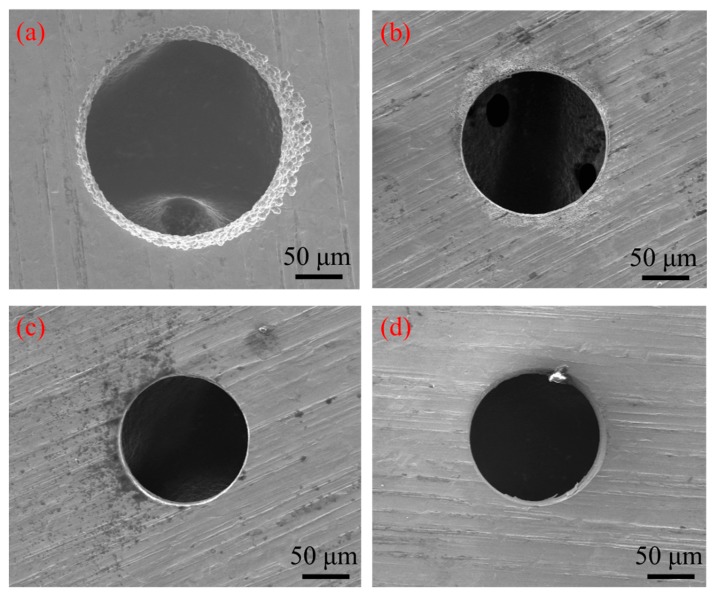
SEM images of inlet profile of micro through-hole fabricated under pulse frequencies of 0 kHz (**a**), 1 kHz (**b**), 50 kHz (**c**), 100 kHz (**d**).

**Figure 19:**
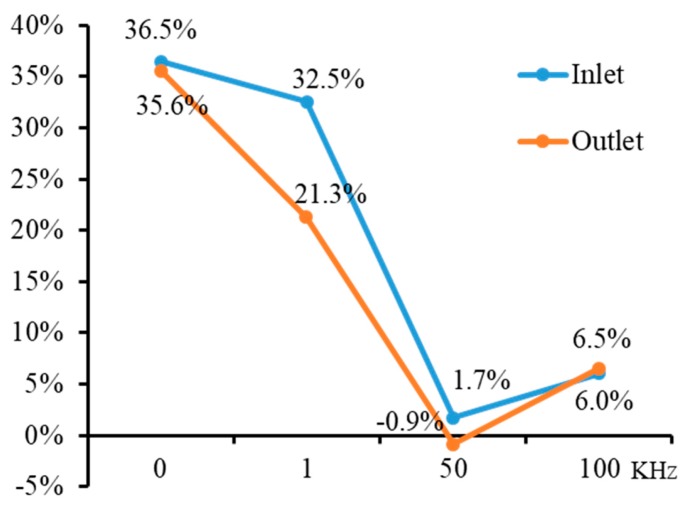
Decline range of inlet and outlet dimension of micro through-hole after using micro helical electrode coated with non-conductive mask.

Figure 17 shows the influence of pulse frequency on the inlet and outlet dimension of micro through-hole fabricated by using micro helical electrode coated with non-conductive mask. Four groups of machining experiments of micro through-holes with the pulse frequencies of 0, 1, 50, and 100 kHz are conducted. The other experimental conditions are 2000 rpm rotating speed, 10 V machined voltage, 20% duty cycle, 1 μm/s feeding speed, 2% NaNO_3_ electrolyte, respectively. The experimental results show that compared with the experimental results obtained by using micro helical electrode without the non-conductive coat, as shown in Figure 6, when using micro helical electrode coated with the non-conductive mask, both the inlet and outlet dimension decrease much more rapidly with the increase of the pulse frequency. Figure 18 shows the SEM images of inlet profile of micro through-hole fabricated under pulse frequencies of 0, 1, 50, and 100 kHz. It can be found that compared with the SEM images of inlet profile of micro through-hole machined under the same pulse frequency in EMM without mask, the machining quality of micro through-hole is improved significantly. Figure 19 shows the decline range of the inlet and outlet dimension of micro through-hole after using micro drill coated with non-conductive mask. The decline range of the inlet and outlet dimension of a micro through-hole is defined by the equations

(1) $$100\%\times \left({\bar{D}}_{inlet}^{}-{\bar{D}}^{\prime }{}_{inlet}\right)/{\bar{D}}_{inlet}^{}$$

(2) $$100\%\times \left({\bar{D}}_{outet}^{}-{\bar{D}}^{\prime }{}_{outet}\right)/{\bar{D}}_{outet}^{}$$

Here, ${\bar{D}}_{inlet}^{}$ is the average diameter of the inlet of micro through-hole machined by using helical electrode without mask, as shown in Figure 6, ${\bar{D}}^{\prime }{}_{inlet}$ is the average diameter of the inlet of micro through-hole machined by using helical electrode with mask, as shown in Figure 17, ${\bar{D}}_{outet}^{}$ is the average diameter of the outlet of micro through-hole machined by using helical electrode without mask, as shown in Figure 6, ${\bar{D}}^{\prime }{}_{outet}$ is the average diameter of the outlet of micro through-hole machined by using helical electrode with mask, as shown in Figure 17.

**Figure 20:**
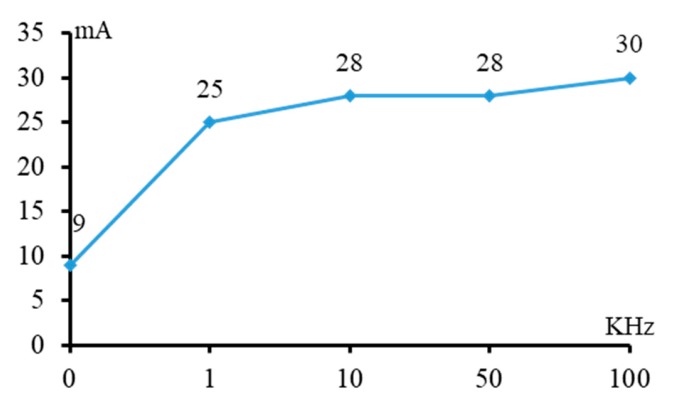
Machining current with different pulse frequencies.

From Figure 19, it can found that when the pulse frequency is low, such as 0 kHz and 1 kHz, the decline range of the inlet and outlet dimension after using micro helical electrode coated with non-conductive mask is significantly large. However, under the condition of high pulse frequency, the decline range of the inlet and outlet dimension after using micro helical electrode coated with non-conductive mask is not obvious. This is because under the condition of pulse frequency, the cathode electrode and anode electrode work like a capacitor and can still transfer the charges between each other, and the non-conductive mask coated on the surface of micro helical electrode cannot totally prevent the stray current. Moreover, according to the basic theory of the capacitor, it can be known that the higher the pulse frequency, the larger the machining current, which is demonstrated by Figure 20. From Figure 20, it can be seen that, under the condition of 0 kHz, the machining current is quite low, and it increases rapidly along with the increase of the pulse frequency and keeps stable when the pulse frequency exceeds 10 kHz. Thus, it can be concluded that non-conductive mask coated on the surface of a helical electrode has good effect on preventing the stray corrosion when the pulse frequency is low and its effect decreases along with the increase of pulse frequency.

## 5. Conclusions

This paper investigates the electrochemical micromachining of micro through-holes by combining micro helical electrode with jetting electrolyte together. Research findings are shown as follows:

1. The experimental and simulation results demonstrate that both the rotating helical electrode and jetting electrolyte can stir and refresh the electrolyte efficiently, improving the machining stability of micro through-hole in EMM. When the rotating speed of a micro helical electrode exceeds a certain value, its influence weakens greatly.
2. Through the optimization of process parameters, micro through-hole with the inlet dimension of 121.6 μm and the outlet dimension of 114.9 μm is obtained successfully through the EMM by using micro helical electrode without non-conductive mask and combining it with the jetting electrolyte, demonstrating that using a micro helical electrode combined with a jetting electrolyte is a much simpler and lower-cost way to improve the machining stability, quality, and accuracy in the EMM of micro through-hole.
3. When using micro helical electrode coated with the non-conductive mask in EMM, both the inlet and outlet dimension of micro through-hole decrease significantly under the condition of low pulse frequency (≤1 KHz). However, under the condition of high pulse frequency, the decline range of the inlet and outlet dimensions of micro through-holes is not obvious.

## Figures and Tables

**Table 1:** Experimental conditions used in electrochemical micromachining (EMM).

Process Parameters	Value
Diameter of helical electrode	100 μm
Rotating speed of spindle	0, 2000, 6000, 10000 rpm
Machined voltage	8, 10, 12, 15, 17 V
Workpiece	Stainless steel (SUS304)
Voltage frequency	0, 1, 10, 50, 100, 500, 700 kHz
Voltage duty	20%, 30%, 40%, 50%
Feeding rate	0.5, 1, 2 μm/s
Electrolyte concentration	2%, 5%, 8%, 11% NaNO_3_ electrolyte
Flow rate of electrolyte	30 mL/min
